# Error in Text

**DOI:** 10.1001/jamahealthforum.2024.5626

**Published:** 2025-01-31

In the Editorial titled “Medicare to Veterans Affairs Cost Shifting—A Challenging Conundrum,”^1^ which published December 27, 2024, there were errors in the text. The first paragraph now reports some 1700 facilities of various types, rather than 1400. Text in the seventh paragraph was also clarified. This article has been corrected.
